# NIRS frequency analysis to evaluate cerebrovascular reactivity after acute brain injury

**DOI:** 10.1117/1.NPh.12.4.045011

**Published:** 2025-12-08

**Authors:** Giulio Bicciato, Ignazio de Trizio, Giovanna Brandi, Jan F. Willms, Emanuela Keller

**Affiliations:** University Hospital Zurich and University of Zurich, Institute of Intensive Care Medicine, Clinical Neuroscience Center, Department of Neurosurgery, Neurocritical Care Unit, Zurich, Switzerland

**Keywords:** near-infrared spectroscopy, acute cerebral injury, neurophysiology, hyperoxia, cerebral autoregulation

## Abstract

**Significance:**

Cerebral autoregulation (CA) relies on cerebrovascular reactivity, and it is often impaired after acute brain injury (ABI), contributing to secondary brain damage. Noninvasive neuromonitoring with near-infrared spectroscopy (NIRS) can detect oscillations in cerebral hemodynamics, which may reflect autoregulatory function and may therefore be a candidate biomarker for CA monitoring.

**Aim:**

We investigated whether changes in the spectral composition of the NIRS signal in ABI under the condition of mild hyperoxia are associated with clinical outcomes in ABI patients.

**Approach:**

Bilateral prefrontal NIRS recording was performed during hyperoxia challenges in mechanically ventilated ABI patients admitted to the neurocritical care unit. Spectral power in low-frequency and very low-frequency oscillations (VLFO) was computed and correlated with clinical scores and functional outcomes (Glasgow Outcome Scale).

**Results:**

Patients with favorable outcomes exhibited increased VLFO during mild hyperoxia with a fraction of inspired oxygen (${\mathrm{FiO}}_{2}$) of 50%, whereas no further rise occurred at ${\mathrm{FiO}}_{2}$ 70%, likely due to oxygen-induced vasoconstriction. In a subgroup of patients with subarachnoid hemorrhage, VLFO changes correlated with clinical severity and independently predicted outcome.

**Conclusions:**

Our preliminary findings in a group of 20 patients suggest a potential role of NIRS in the development of individualized neuromonitoring strategies for brain-injured patients.

## 1 Introduction

Cerebral autoregulation (CA) is the ability of the brain to maintain stable cerebral blood flow (CBF) despite fluctuations in cerebral perfusion pressure. This relies on cerebrovascular reactivity (CVR), the mechanism of regulating CBF through vasodilation and vasoconstriction in response to different physiological and pathological stimuli. The impairment of this mechanism has been linked to poor neurological outcomes in various acute brain injuries, including traumatic brain injury, subarachnoid hemorrhage, and stroke.^1^^–^^3^ Monitoring CA in real time is of great relevance in a neurocritical care setting, and several invasive and noninvasive methods have already been investigated.

Near-infrared spectroscopy (NIRS) provides continuous measurements of local cerebral oxygenation. Because cerebral oxygenation reflects the balance between oxygen delivery and consumption, NIRS-derived measurements can serve as an indirect surrogate of CVR. However, although promising, clinical implementation of NIRS remains limited. A few NIRS-derived indices, such as the total hemoglobin index (THI), the cerebral oxymetry index (COx), or hemoglobin volume index (HVx) have garnered relevant scientific support.^4^^–^^6^ These indices reflect the correlation between NIRS-derived parameters reflecting changes in cerebral oxygenation and continuously recorded arterial blood pressure. However, interpretation of NIRS signals remains challenging as they are influenced by extracerebral structures such as the scalp, bone, and skin, whose own vascularization introduces noise that overlaps with cerebral signals. Moreover, the reliability of these indices is further questioned by recent studies showing paradoxical findings, such as normal cerebral saturation in patients diagnosed with brain death,^7^ or inconsistencies between COx and invasive assessment of CA with intracranial pressure measurement or pressure reactivity index.^8^ Some earlier work indicates that the spectral composition of the NIRS signal may influence the reliability of autoregulation indices, with better performance observed when the signal has higher spectral power in the very low-frequency band.^9^^,^^10^ However, it remains unexplained why the very low-frequency component of the NIRS signal varies among different subjects, and to the best of our knowledge, it has not been well characterized how different pathological conditions may influence these oscillations. Therefore, a deeper understanding of the neurophysiological basis underlying the NIRS signal is essential to develop a conceptual framework for interpreting these measurements and translating them into clinical practice. Spontaneous hemodynamic oscillations attributed to cerebral vasomotion are detectable via NIRS, transcranial Doppler (TCD), and functional magnetic resonance imaging (fMRI) with spectral peaks in the low- (LFO, ∼0.1  Hz) and very low-frequency (VLFO, ∼0.04  Hz) ranges.^11^^–^^16^ However, it is important to note that signals detected within very-low-frequency oscillations (VLFO) and low-frequency oscillations (LFO) spectral bands are also influenced by systemic vascular oscillations. For example, at the same frequency as LFOs, systemic Mayer waves can be observed in arterial blood pressure, likely under sympathetic autonomic control.^17^^,^^18^ Nevertheless, it has been extensively demonstrated that within these frequency bands, a relevant portion of the signal originates specifically from cerebral sources.^19^^,^^20^ In fact, LFOs are thought to reflect local vasomotor response to neuronal activity,^11^^,^^21^^–^^23^ whereas VLFOs reflect oscillations in large arterioles under neurogenic innervation. Changes in VLFO amplitude have been described in relation to vasomotor response to different frequencies of respiration changing the arterial partial pressure of carbon dioxide (${\mathrm{PaCO}}_{2}$) level.^24^ Both LFO and VLFO have been described to decrease with age and cognitive decline, which implicitly suggests that these oscillations represent physiological features typically expected in a healthy brain.^25^^–^^27^

The aim of the present study is to advance the development of an NIRS-based biomarker for CVR by characterizing, so far as possible, a cerebral-specific signal distinguished from extracerebral noise. Although separating these components is a major challenge, our experimental paradigm is designed to highlight oxygenation changes across patients with varying degrees of brain injury. Comparing patients with widespread damage and poor prognosis may help reveal physiological mechanisms expected in healthy subjects, which are otherwise masked by noise from extracerebral factors. We hypothesized that spontaneous hemodynamic oscillations respond differently to physiological stimuli depending on the integrity of cerebral vasomotion. Specifically, we would expect patients with milder brain injury and better prognosis to exhibit greater amplitude variations in these oscillations compared with patients with more severe injury. To test this, we introduced variations in the fraction of inspired oxygen (${\mathrm{FiO}}_{2}$). Prolonged hyperoxia is generally avoided in acute brain injury (ABI) due to free radical production and cerebral vasoconstriction.^28^^–^^30^ However, short-term mild hyperoxygenation (${\mathrm{FiO}}_{2}$ up to 70%) is commonly used during suctioning or transport without major safety concerns.^31^^,^^32^ The expected effect of hyperoxia on the NIRS signal in healthy brain tissue can be postulated to result, on the one hand, from a relative increase in the $O_{2}\mathrm{Hb}$ signal, and on the other hand, from potential vasoconstrictive mechanisms that hyperoxia may trigger beyond a certain threshold.^33^^,^^34^ We postulate that patients with more severe brain injury exhibit altered cerebral vasomotion; therefore, their response to different ${\mathrm{FiO}}_{2}$ would be less pronounced, whereas the extracerebral signal would remain unchanged. The first purpose of this study was to characterize the physiological mechanisms of CVR, as reflected by changes in LFO and VLFO, during hyperoxia. The secondary objective was to evaluate whether the hyperoxic response of these oscillations correlates with functional outcomes at 6 and 12 months.

## 2 Materials and Methods

### 2.1 Participants

The study included 20 mechanically ventilated patients admitted to the Neurocritical Care Unit (NCCU) of the University Hospital Zurich with ABI. Exclusion criteria were age≤18 years, and hemodynamic or respiratory instability at the time of the measurement, based on the clinical judgement of the treating physicians. Given the exploratory nature of the study, patients were enrolled through convenience sampling. The study was approved by the Cantonal Ethics Committee of the Canton of Zurich, Switzerland (BASEC ID 2019-00345). The study protocol was in accordance with the Declaration of Helsinki. Written assent was given by legal representatives, as all patients were deemed incapable of providing informed consent.

### 2.2 Demographics

The mean age of the patients was 65 years (±12). Of them, 7 were males, and 13 were females. The underlying pathologies comprised intracerebral hemorrhage (ICH) in 5 patients, ischemic stroke (IS) in 4 patients, and aneurysmal subarachnoid hemorrhage (aSAH) in 11 patients. Lesions were lateralized to the right or left hemisphere in 15 patients, whereas the other patients presented with bilateral brain lesions. Table 1 provides an overview of the demographic, clinical, and outcome characteristics of the study population. Table S1 in the Supplementary Material provides an overview of further clinical characteristics of the patients, such as days intercurred as the index ABI, presence of sedation, and vasoactive.

**Table 1:** Including patient characteristics, underlying pathology (ICH = intracerebral hemorrhage, IS = ischemic stroke, aSAH = aneurysmal subarachnoid hemorrhage), lateralization of the lesion, Glasgow Outcome Score (GOS) at 6 and 12 months. In addition, the Hunt and Hess (H&H) and World Federation of Neurological Societies (WFNS) grades are included for patients with aSAH.

Patient	Age	Sex	Underlying pathology	Lateralization	Hunt and Hess	WFNS	GOS 6 months	GOS 12 months
1	60	F	ICH	Right			3	3
2	93	F	IS	Right			3	1
3	69	M	IS	Right			3	3
4	71	F	ICH	Right			3	3
5	65	F	ICH	Left			4	5
6	46	M	IS	Left			4	5
7	72	M	ICH	Left			4	4
8	48	M	aSAH	Bilateral	4	4	1	1
9	71	F	aSAH	Bilateral	1	1	5	5
10	55	M	aSAH	Right	5	5	1	1
11	70	F	IS	Right			3	1
12	64	F	ICH	Right	5	5	1	1
13	54	F	aSAH	Right	4	4	2	2
14	62	M	aSAH	Bilateral	3	2	4	4
15	42	F	aSAH	Right	3	2	5	5
16	78	F	aSAH	Left	1	1	3	3
17	74	F	aSAH	Bilateral	3	2	1	1
18	54	F	aSAH	Left	4	4	4	4
19	76	M	aSAH	Right	3	4	1	1
20	67	F	aSAH	Bilateral	3	4	5	4

### 2.3 Clinical Assessment of Patients

Mortality and Glasgow outcome score (GOS) were extracted at 6 and 12 months from electronic medical records. As subarachnoid hemorrhage (SAH) was the most common diagnosis in our cohort (n=11 patients), additional clinical severity scores were collected: Hunt & Hess (H&H) and World Federation of Neurosurgical Societies score (WFNS). Catecholamine therapy was categorized into three groups: 1) No catecholamine, 2) Low dose noradrenaline (<0.1  mcg/Kg/min), and 3) higher dose (≥0.1  mcg/Kg/min) of noradrenaline or medication with dobutamine. Deep sedation was defined as intravenous administration of propofol, midazolam, ketamine, thiopental, and sufentanyl. Patients receiving only low-dose clonidine or dexmedetomidine were considered not deeply sedated. Cerebral computed tomography (CT) and MRI were reviewed for all patients to assess the lateralization of brain lesions. Based on the imaging findings, patients were categorized into three groups: left-lateralized lesions, right-lateralized lesions, and global or bilateral lesions.

### 2.4 fNIRS Measurement

NIRS-data acquisition was performed with OXYMON Mk III and Oxysoft (version 3.0, 103.3, Artinis Medical Systems B.V., Elst, The Netherlands). Two optodes were applied bilaterally on the forehead as already described in previous works.^35^ The inter-optode distance, i.e., the distance between the main light source and the light detector, was 35 mm. The sampling frequency was 25 Hz. A differential pathlength factor (DPF) of 6 was used. Changes in oxygenated hemoglobin ($O_{2}\mathrm{Hb}$) were automatically computed by Oxysoft software according to the modified Lambert–Beer law.

### 2.5 Experimental Protocol

All patients had a baseline arterial partial oxygen pressure $({\mathrm{PaO}}_{2})>11\;\mathrm{kPa}$ with a 30% to 35% ${\mathrm{FiO}}_{2}$. The experimental protocol consisted of three measurement blocks, each lasting 5 min (Fig. S1 in the Supplementary Material). Given the VLFO and LFO frequencies of interest, a 5-min recording was considered the minimum duration sufficient to capture at least three cycles of oscillations around 0.01 Hz and to provide adequate spectral resolution to separate the different frequency bands. At the same time, limiting each experimental block to 5 min (i.e., 10 min of cumulative mild hyperoxygenation) ensured that the protocol remained within safe exposure limits.

1. Baseline (${\mathrm{FiO}}_{2}$ 30% to 35%): routine ventilatory settings maintained, with ${\mathrm{PaO}}_{2}>11\;\mathrm{kPa}$.
2. Oxygen Challenge (${\mathrm{FiO}}_{2}$ 50%): ${\mathrm{FiO}}_{2}$ increased to 50%.
3. Oxygen Challenge (${\mathrm{FiO}}_{2}$ 75%): ${\mathrm{FiO}}_{2}$ further increased to 75%.

NIRS was recorded at each ${\mathrm{FiO}}_{2}$ level to evaluate the effects of increasing oxygen concentrations. The minute volume of ventilation was maintained constant throughout the experiment in order to prevent variations in end tidal carbon dioxide (${\mathrm{EtCO}}_{2}$) levels. Mean blood pressure was kept stable throughout the experiment.

### 2.6 Collection of Systemic Vital Parameters

Mean arterial pressure (MAP), heart rate (HR), oxygen saturation (${\mathrm{SpO}}_{2}$), and respiratory rate (RR) were continuously monitored and recorded throughout the experiment. All signals were acquired using the Philips Intellivue system (Philips Medical systems, Boeblingen, Germany) and transmitted to a CNS Data Collector (Moberg ICU Solutions, Ambler, Philadelphia, USA). Signals were sampled at 1.024 Hz. Data were collected and exported using our proprietary data collection platform ICU Cockpit.^36^

### 2.7 Processing of fNIRS-Data and Statistical Analysis

Signal processing and statistical analyses were conducted in MATLAB_R2024b (Mathworks, Natick MA, USA). For the statistical analysis, $O_{2}\mathrm{Hb}$ signal was used, due to its better signal-to-noise ratio compared with deoxygenated hemoglobin (HHb).^35^^,^^37^ The $O_{2}\mathrm{Hb}$ signal was segmented according to the three experimental conditions (baseline, ${\mathrm{FiO}}_{2}$ 50%, ${\mathrm{FiO}}_{2}$ 70%). Similar to previous works,^35^^,^^38^ we defined two distinct frequency bands: LFO (0.04 to 0.2 Hz) and VLFO (0.008 to 0.04 Hz). Changes in LFO and VLFO were assessed by calculating the ratio of mean spectral power between subsequent experimental blocks: from the baseline (B) to ${\mathrm{FiO}}_{2}$ 50% (B → ${\mathrm{FiO}}_{2}$ 50%) and from ${\mathrm{FiO}}_{2}$ 50% to ${\mathrm{FiO}}_{2}$ 70% (${\mathrm{FiO}}_{2}$ 50% → ${\mathrm{FiO}}_{2}$ 70%). The ratio was then transformed with the natural logarithm (Ln) as follows:

$${\mathrm{LFO}\;\text{Ratio}}_{B\to \mathrm{FiO}2\;50\%}=Ln(\frac{\text{mean}(\mathrm{LFO}.{\text{Power}}_{\mathrm{FiO}2\;50\%})}{\text{mean}(\mathrm{LFO}.{\text{Power}}_{B})}),\;{\mathrm{LFO}\;\text{Ratio}}_{\mathrm{FiO}2\;50\%\to \mathrm{FiO}2\;70\%}=Ln(\frac{\text{mean}(\mathrm{LFO}.{\text{Power}}_{\mathrm{FiO}2\;70\%})}{\text{mean}(\mathrm{LFO}.{\text{Power}}_{\mathrm{FiO}2\;50\%})}),$$

$${\mathrm{VLFO}\;\text{Ratio}}_{B\to \mathrm{FiO}2\;50\%}=Ln(\frac{\text{mean}(\mathrm{VLFO}.{\text{Power}}_{\mathrm{FiO}2\;50\%})}{\text{mean}(\mathrm{VLFO}.{\text{Power}}_{B})}),\;{\mathrm{VLFO}\;\text{Ratio}}_{\mathrm{FiO}2\;50\%\to \mathrm{FiO}2\;70\%}=Ln(\frac{\text{mean}(\mathrm{VLFO}.{\text{Power}}_{\mathrm{FiO}2\;70\%})}{\text{mean}(\mathrm{VLFO}.{\text{Power}}_{\mathrm{FiO}2\;50\%})}).$$

A logarithmic transformation was performed, as in our previous work, primarily to stabilize variance and to improve graphical representation, particularly for group comparisons. After applying the Lilliefors and Jarque–Bera tests, the transformed data were found to be compatible with a normal distribution.

As no gold standard exists to establish a definitive noninvasive measure of intact or impaired autoregulation, a preliminary analysis was conducted in the subgroup of 8 patients with the most favorable outcome after 12 months, defined by a GOS of 4 or 5. Within this sample, changes in LFO and VLFO were observed as the best approximation of physiological processes.

Subsequently, variations in LFO and VLFO were examined in the entire cohort, stratifying patients based on clinical outcomes (GOS). Associations with GOS were assessed using Spearman’s rank correlation test. If a significant association was found, patients were grouped into those with unfavorable outcomes (GOS 1 to 2) and those with favorable outcomes (GOS 3 to 5). Between-group differences were tested using Wilcoxon rank-sum test.

In the subgroup of patients with lateralized brain lesions, to assess for hemispheric difference in LFO and VLFO, comparisons were made between the ipsilateral and contralateral hemispheres with the Wilcoxon rank-sum test.

In a post hoc analysis, associations between VLFO changes (VLFO-Ratio) and GOS after 6 and 12 months were further analyzed using multivariate regression models. Covariates included: age, sex, deep sedation, and therapy with catecholamines. Within the subgroup of 11 patients with aSAH, the association between VLFO-Ratio and the clinical severity scores (H&H and WFNS) was evaluated using Spearman’s rank test. In this specific group, the multivariate regression model included age, sex, deep sedation, catecholamines, along with H&H and WFNS scores, to determine whether VLFO-Ratio serves as an independent predictor of GOS at 6 and 12 months.

The statistical significance threshold was set at p<0.05.

## 3 Results

1. **Preliminary analysis, seeking the most physiological response.**

**Fig. 1:**
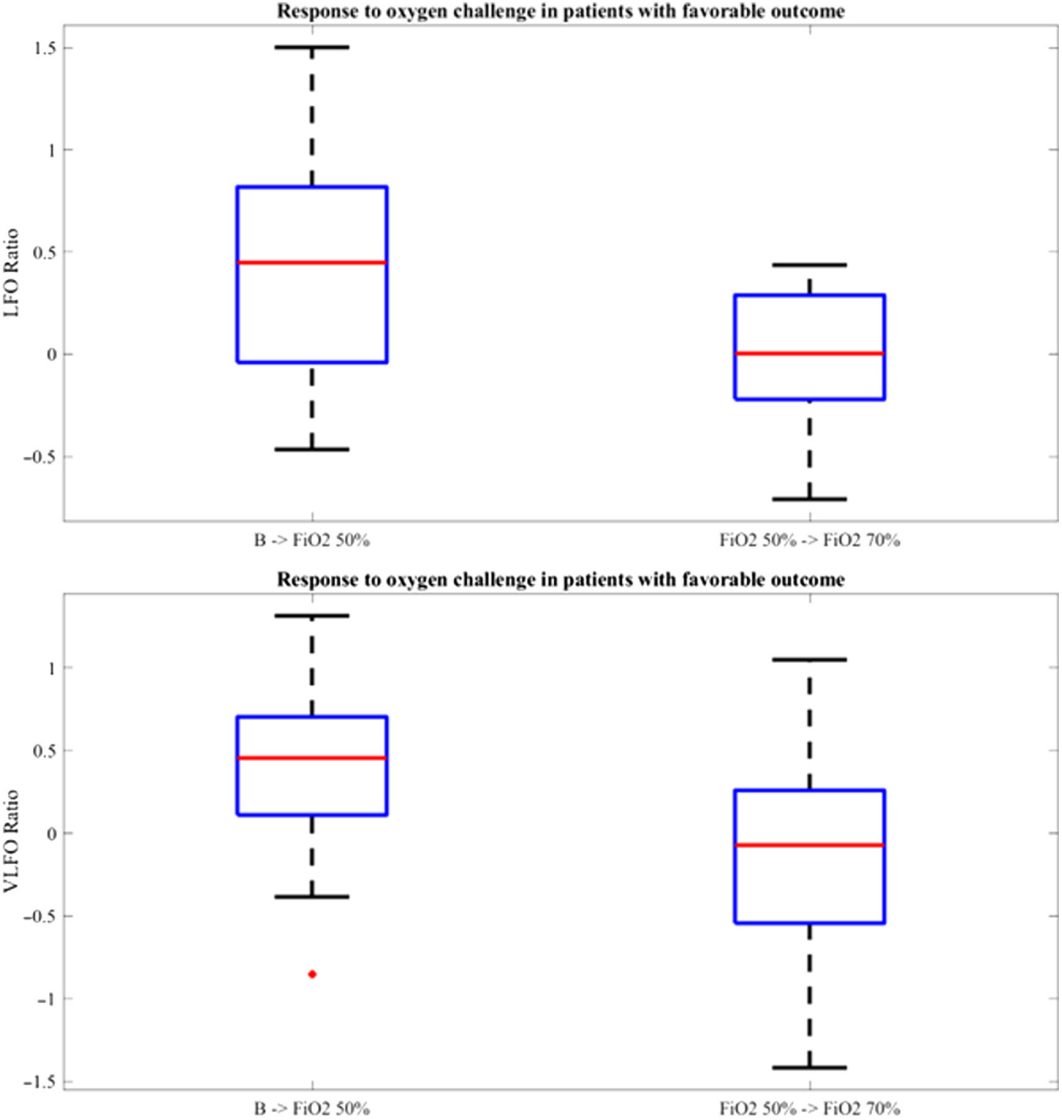
Boxplot illustrating LFO ratio (above) and VLFO ratio (below) values after increasing ${\mathrm{FiO}}_{2}$ to 50% from baseline ($B\to {\mathrm{FiO}}_{2}$ 50%) and from ${\mathrm{FiO}}_{2}$ 50% to 70% (${\mathrm{FiO}}_{2}$ $50\%\to {\mathrm{FiO}}_{2}$ 70%) in the 8 patients with GOS≥4.

A preliminary analysis was conducted in the subgroup of the 8 patients with the most favorable outcomes (GOS≥4), the results are displayed in Fig. 1.

a. LFO

An increase in LFO spectral power was observed after raising ${\mathrm{FiO}}_{2}$ to 50% ($\mathrm{LFO}.{\text{Ratio}}_{B\to \mathrm{FiO}2\;50\%}$ mean 0.42, STD 0.57). However, no further increase was detected when ${\mathrm{FiO}}_{2}$ was elevated to 70% ($\mathrm{LFO}.{\text{Ratio}}_{\mathrm{FiO}2\;50\%\to \mathrm{FiO}2\;70\%}$ mean −0.03, STD 0.37). $\mathrm{LFO}\text{-}{\text{Ratio}}_{B\to \mathrm{FiO}2\;50\%}$ was significantly higher than $\mathrm{LFO}\text{-}{\text{Ratio}}_{\mathrm{FiO}2\;50\%\to \mathrm{FiO}2\;70\%}$ (Wilcoxon signed rank test, rank statistic = 327, p<0.05).

b. VLFO

An increase in VLFO spectral power was observed after raising ${\mathrm{FiO}}_{2}$ to 50% ($\mathrm{LFO}\text{-}{\text{Ratio}}_{B\to \mathrm{FiO}2\;50\%}$ mean 0.40, STD 0.55). However, no further increase was detected when ${\mathrm{FiO}}_{2}$ was elevated to 70% ($\mathrm{LFO}.{\text{Ratio}}_{\mathrm{FiO}2\;50\%\to \mathrm{FiO}2\;70\%}$ mean −0.12, STD 0.64). $\mathrm{VLFO}\text{-}{\text{Ratio}}_{B\to \mathrm{FiO}2\;50\%}$ was significantly higher than $\mathrm{VLFO}\text{-}{\text{Ratio}}_{\mathrm{FiO}2\;50\%\to \mathrm{FiO}2\;70\%}$ (Wilcoxon signed rank test, rank statistic = 328, p<0.05).

2. **LFO during oxygen challenge**

Oxygen challenge: ${\mathrm{FiO}}_{2}$ 50% and 70%

No significant association was found between GOS at 6 and 12 months and LFO-Ratio after increasing the ${\mathrm{FiO}}_{2}$ up to 50% and 70% (Spearman’s rank correlation rho=−0.0399, p=0.88, and rho=−0.3506, p=0.1297, respectively).

**Fig. 2:**
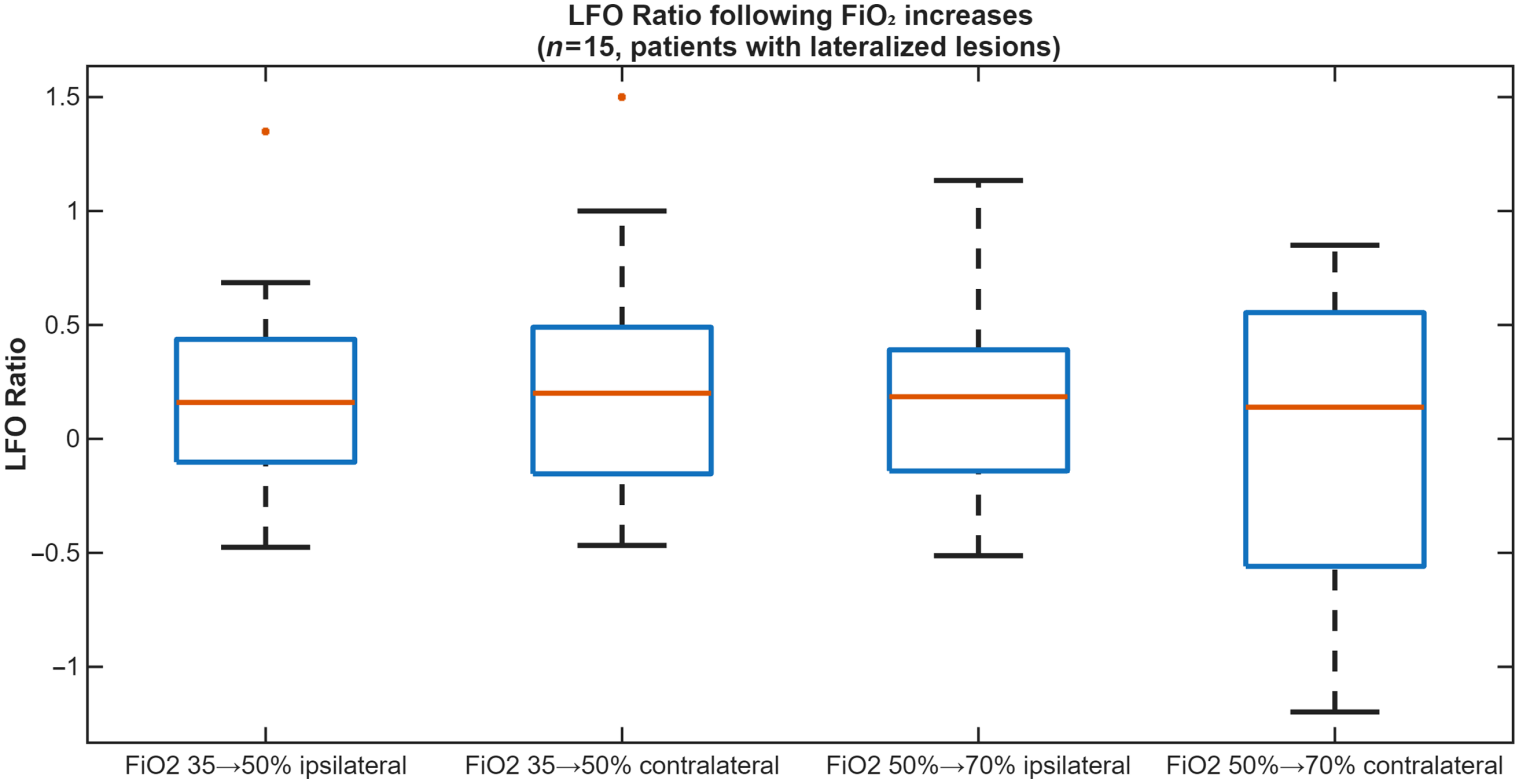
Boxplot showing LFO ratio values following ${\mathrm{FiO}}_{2}$ increases from baseline to 50% ($B\to {\mathrm{FiO}}_{2}$ 50%) and from 50% to 70% (${\mathrm{FiO}}_{2}$ $50\%\to {\mathrm{FiO}}_{2}$ 70%), analyzed by ipsilateral versus contralateral hemisphere in a subgroup of 15 patients with lateralized brain lesions. No significant differences in LFO response were observed.

In the subgroup of 15 patients with lateralized brain lesions, no significant differences in LFO response were observed between the hemisphere ipsilateral and contralateral to the lesion (Fig. 2).

**VLFO during oxygen challenge**
  a. Oxygen challenge: ${\mathrm{FiO}}_{2}$ 50%

**Fig. 3:**
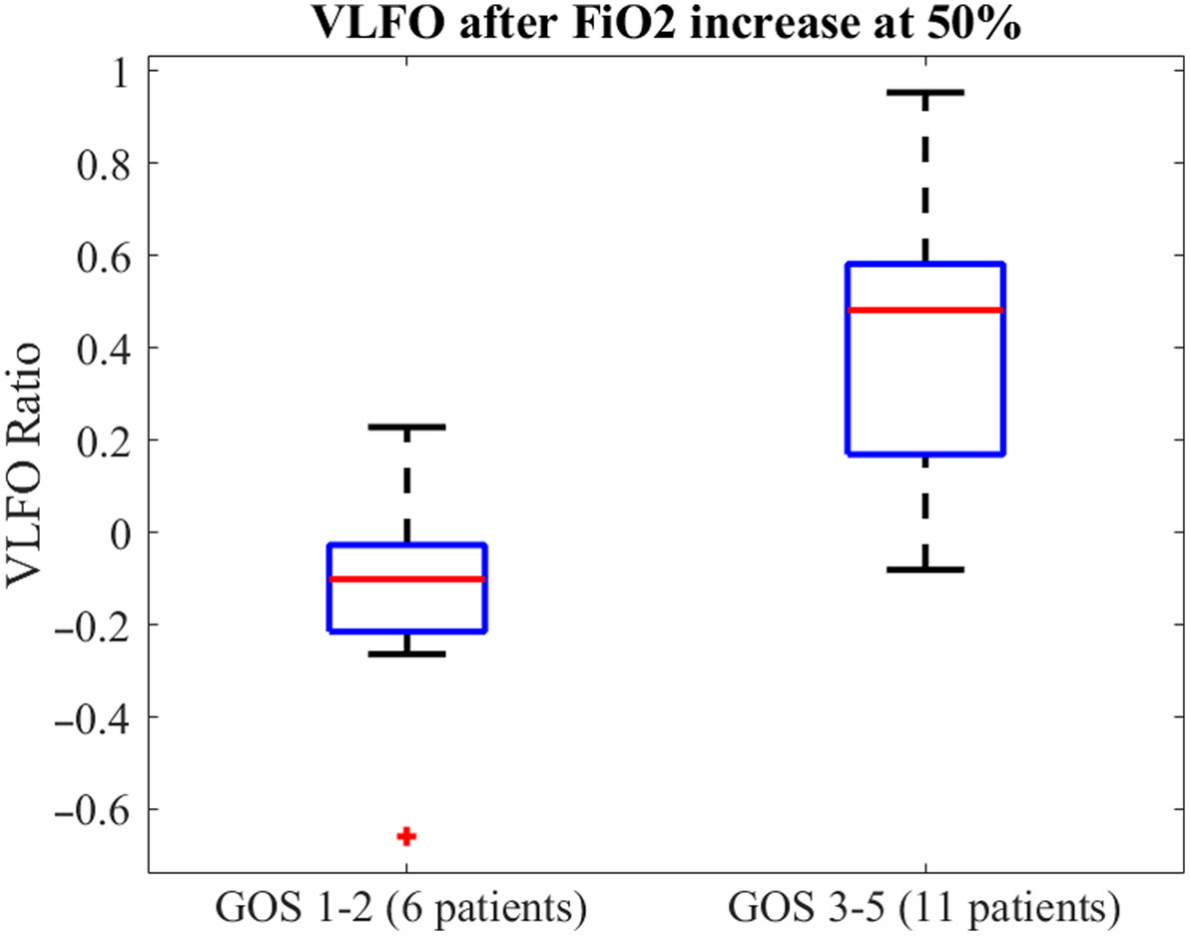
Boxplot comparing VLFO ratio values following an increase in ${\mathrm{FiO}}_{2}$ from baseline to 50% in 6 patients with an unfavorable outcome [defined as a Glasgow Outcome Scale (GOS) score of 1 to 2 at 12 months] versus 11 patients with a more favorable outcome (GOS 3 to 5). VLFO ratio in patients with unfavorable outcome was significantly lower (ranksum = 26, p=0.003).

A moderate positive correlation was observed between the increase in VLFO spectral power with ${\mathrm{FiO}}_{2}$ 50% and GOS at 6 months (Spearman’s rank, rho = 0.48, p=0.03) and at 12 months (Spearman’s rank, rho = 0.59, p=0.0121). After grouping the patients into groups with unfavorable (GOS 1 to 2) and favorable outcomes (GOS 3 to 5), patients with favorable outcomes showed significantly higher VLFO-Ratio compared with patients with unfavorable outcomes (ranksum = 26, p=0.003) (Fig. 3).

In the subgroup of 15 patients with lateralized lesions, no significant differences in VLFO changes were observed when comparing the hemispheres ipsilateral and contralateral to the lesion (Wilcoxon signed rank test, rank statistic = 209, p=0.34).

b. Oxygen Challenge: ${\mathrm{FiO}}_{2}$ 70%

No significant association was observed between GOS and VLFO-Ratio after increasing ${\mathrm{FiO}}_{2}$ to 70% (Spearman’s rank correlation rho=−0.2105, p=0.37). In the subgroup of 15 patients with lateralized brain lesions, slightly higher VLFO-Ratio values were noted in the hemisphere contralateral to the lesion compared with the ipsilateral hemisphere. However, this difference did not reach statistical significance (Wilcoxon signed rank test, rank statistic = 189, p=0.07).

3. **Post-hoc analysis on VLFO-Ratio**

In the multivariate regression analysis on the entire group of patients – including age, sex, deep sedation or catecholamines – VLFO-Ratio did not significantly predict outcome after 6 months (F-statistic p=0.185) but was an independent predictor of outcome at 12 months (p<0.01). In the subgroup of 11 patients with SAH, VLFO-Ratio was significantly associated with both H&H and WFNS scores (Spearman’s rank correlation rho=−0.6421, p<0.05, and rho=−0.6228, p<0.05, respectively). In the multivariate regression analysis, including the two clinical scores as well as age, sex, deep sedation or catecholamines, VLFO-Ratio was an independent predictor of GOS at 6 (p<0.05) and 12 months (p<0.05). The results are presented in Tables S2–S5 in the Supplementary Material.

4. **Correlation with sedation and presence of catecholamine**

No differences in VLFO-Ratio were found after direct comparison between sedated and nonsedated patients. No differences were found in association with the presence or absence of catecholamines.

## 4 Discussion

The aim of this study was to explore potential biomarkers of CVR using frequency-domain analysis of NIRS signals during a controlled oxygen challenge. Given the known influence of extracerebral tissues on NIRS signals, our experimental protocol was specifically designed to isolate and enhance signals of cerebral origin.

Compared with patients with an unfavorable neurological outcome, those with a favorable outcome showed a significant increase in VLFO in response to a controlled oxygen challenge, specifically during mild hyperoxia (${\mathrm{FiO}}_{2}$ 50% from a baseline of 30% to 35% in patients with adequate baseline oxygenation). A post hoc analysis in a subgroup of patients with SAH showed that VLFO changes correlated with clinical severity scores (Hunt and Hess and WFNS) and served as independent predictors of outcome in multivariate models. No further increase in VLFO was observed when ${\mathrm{FiO}}_{2}$ was raised to 70%. This plateau effect may be attributed to hyperoxia-induced vasoconstriction, which limits additional oxygen delivery once cerebral oxygenation exceeds physiological thresholds.

Taken together, these findings suggest that VLFO response to controlled oxygen challenge may serve as a physiologically grounded and clinically relevant marker of cerebral autoregulation. However, the interpretation of these results remains challenging and requires further validation. Nonetheless, we can propose some speculative hypotheses to guide their interpretation. Some evidence suggests that VLFO may arise from oscillations in larger vessels. Similar oscillations have been observed in CBF measured by TCD, in blood-oxygen-level-dependent (BOLD) fMRI, and in jugular bulb cerebral hemoglobin oxygenation measurement.^39^ Moreover, VLFO frequency range overlaps with Lundberg B waves. These are slow, noncardiac rhythmic oscillations of intracranial pressure (ICP) occurring at approximately 0.5 to 2.0 cycles per minute.^40^ Although already detectable in healthy individuals, the B waves’ amplitude increases with elevated ICP,^41^ possibly due to reduced intracranial compliance, which amplifies the transmission of vascular vasomotion into intracranial pressure fluctuations.^42^ Therefore, VLFO measured with NIRS may be capturing the “vascular engine” of B waves. A speculative hypothesis is that the VLFO increase observed during mild hyperoxia may resemble the behavior of a contrast agent, which propagates in viable and perfused brain regions, whereas it does not appear in areas with reduced perfusion or impaired autoregulation. Specifically, an increase in oxygenated hemoglobin amplifies intrinsic vasomotor activity within the VLFO range only if microvascular function is preserved. Conversely, in extensively damaged tissue, such an effect may be absent, resulting in missing VLFO responses. This interpretation is consistent with the lack of further VLFO increase at higher ${\mathrm{FiO}}_{2}$ levels in patients with favorable outcomes, where intact autoregulatory mechanisms may have induced vasoconstriction. This is in line with existing evidence showing that hyperoxia in ABI patients does not improve neurological outcomes and may even increase the risk of oxygen toxicity.^28^^,^^43^^–^^45^

Although increases in LFO during hyperoxia may act as a potential confounder in functional NIRS studies based on LFO analysis, we did not observe any association between LFO changes and clinical outcome, nor any hemispheric differences in patients with lateralized brain lesions. This lack of association may be explained by several factors. LFOs are thought to originate from vasomotion of terminal cerebral arterioles and may partially reflect neurovascular coupling.^21^^,^^22^^,^^46^^,^^47^ Although LFO can also be detected during resting state recording,^25^ their amplitude variations in the absence of functional stimulation may be too low to overcome extracerebral signal contamination. By contrast, VLFO typically exhibits higher spectral power than LFO, potentially resulting in a better signal-to-noise ratio relative to extracerebral signals. Therefore, differences between groups may be more easily detectable.

Despite these promising findings, several limitations must be acknowledged. First, this was an exploratory study with a relatively small sample size and heterogeneous patient population. In particular, the results in the subgroup of patients with SAH are based on a small cohort of 11 patients. Although they show promising internal consistency with validated clinical severity scores, these findings should be interpreted with caution and require validation in larger cohorts. The oxygen challenge was not standardized across all patients in terms of timing relative to injury onset or sedation depth, which may introduce variability in individual responses. Finally, although associations with clinical outcomes were found, we can only provide a speculative explanation of these findings and further studies are needed to establish mechanistic links.

Future research should aim to validate these findings in larger, more homogenous cohorts and explore the relationship between VLFO and established invasive markers of cerebral physiology, such as intracranial pressure and partial pressure of brain tissue oxygen.

## 5 Conclusion

This study provides preliminary evidence that VLFO in the NIRS signal, particularly their response to slight increases in inspired oxygen, may serve as a promising noninvasive biomarker of cerebral autoregulation in neurocritical care patients. Our data support the notion that inducing hyperoxia in ABI patients does not provide additional benefits in terms of cerebral tissue oxygenation. Further validation of our findings could be obtained through simultaneous measurements of NIRS with invasive ICP and partial pressure of oxygen in brain tissue (${\mathrm{PbtO}}_{2}$) monitoring. In this context, future work should focus on elucidating the neurophysiological mechanisms underlying VLFO and clarifying their relationship with invasive measures of intracranial pressure and autoregulation.

## Supplementary Material

10.1117/1.NPh.12.4.045011.s01

## Data Availability

The datasets and code used during the current study are available from the corresponding author on reasonable request.
