# Supplementary material for: NIRS frequency analysis to evaluate cerebrovascular reactivity after acute brain injury
Source: Neurophotonics. 2025 Dec 8;12(4):045011. doi: 10.1117/1.NPh.12.4.045011 (PMC12685250; doi:10.1117/1.NPh.12.4.045011)
Supplement: Supplementary file 1 [file NPh_012_045011_SD001.pdf]

Supplementary Material

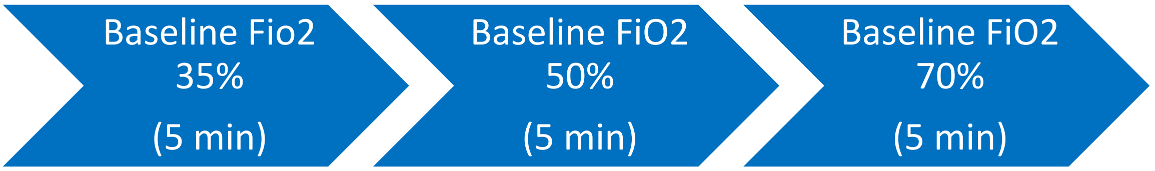

Figure S1 Representation of the three subsequent experimental blocks. During the recording a member of the study team checked for external interactions with the subject (e.g. no nursing intervention or manipulation of the ventilation setting were allowed).

Table S1 including clinical characteristics of the patients: days intercurred since the acute brain injury (ABI), sedation status (0 = no sedation, 1 = under sedation), and vasopressor use (0 = no catecholamine, 1 = noradrenaline < 0.1 mcg/Kg/min, 2 = noradrenaline ≥ 0.1 mcg /Kg/min or dobutamine).

Patient	Days after ABI	Deep sedation	Vasoactiva
**1**	8	no	0
**2**	1	yes	1
**3**	9	no	1
**4**	45	no	2
**5**	7	no	0
**6**	4	yes	1
**7**	8	no	2
**8**	18	no	1
**9**	9	yes	0
**10**	10	yes	1
**11**	18	no	1
**12**	35	no	2
**13**	2	yes	1
**14**	22	yes	2
**15**	5	yes	2
**16**	22	no	2
**17**	2	yes	2
**18**	4	yes	1
**19**	4	no	1
**20**	9	yes	2

Table S2 Estimated coefficients from the linear regression model evaluating the relationship between patient characteristics (Age, Sex, Deep Sedation, and Catecholamines) and VLFO spectral power with the Glasgow Outcome Scale after 6 months. Estimated effect size (Estimate), standard error (SE), t-statistic (tStat), and p-value (pValue) for is shown each variable. Notably, VLFO is the only significant predictor ($p < 0.05$), however the model explains 38.7% of the variance ($R^2 = 0.387$) and fails to reach the significance threshold (F-statistic p-value = 0.185)

Variable	*Estimate*	*SE*	*t-Statistic*	*p-Value*
(Intercept)	*3.1238*	*1.8709*	*1.6697*	*0.11717*
Age	*-0.0044249*	*0.025651*	*-0.1725*	*0.86551*
Sex	*-1.1054*	*0.65988*	*-1.6751*	*0.11609*
Deep Sedation	*0.64752*	*0.6041*	*1.0719*	*0.30191*
Catecholamines	*-0.11502*	*0.41681*	*-0.27596*	*0.78661*
VLFO	*1.9758*	*0.81977*	*2.4102*	*0.030269*

Table S3 Estimated coefficients from the linear regression model evaluating the relationship between patient characteristics (Age, Sex, Deep Sedation, and Catecholamines) and VLFO spectral power with the Glasgow Outcome

Scale after 12 months. The table includes the estimated effect size (Estimate), standard error (SE), t-statistic (tStat), and p-value (pValue) for each predictor. VLFO is the only statistically significant variable ($p < 0.05$). The model explains approximately 60.8% of the variance ($R^2 = 0.608$, Adjusted $R^2 = 0.468$). The F-statistics indicate the model is significant overall ($p < 0.01$).

Variable	*Estimate*	*SE*	*t-Statistic*	*p-Value*
(Intercept)	*4.2648*	*1.5679*	*2.7201*	*0.01659*
Age	*-0.027779*	*0.021497*	*-1.2922*	*0.2172*
Sex	*-1.0905*	*0.55301*	*-1.9719*	*0.068708*
Deep Sedation	*0.402*	*0.50627*	*0.79405*	*0.44042*
Catecholamines	*-0.055734*	*0.34931*	*-0.15956*	*0.87551*
VLFO	*2.8331*	*0.68701*	*4.1238*	*0.001033*

Table S4 linear regression model evaluating the relationship between Age, Sex, Deep Sedation, Catecholamine, Hunt&Hess, WFNS and VLFO.Ratio with the Glasgow Outcome Scale after 6 months. The model explains nearly all the variance ($R^2 = 0.996$,Adjusted $R^2 = 0.98$), with an overall significant fit (F-statistic = 61.1, $p = 0.0162$). VLFO.ratio appears to be an independent predictor of GOS after 6 months ($p = 0.0050$), even when controlling for possible confounders included in the model.

**Variable**	**Estimate**	**SE**	**tStat**	**pValue**
(Intercept)	6.602	1.101	5.9962	0.026704
Age	-0.028715	0.022329	-1.286	0.32724
Sex	-1.1209	0.33877	-3.3086	0.080476
Deep Sedation	2.8135	0.36472	7.7143	0.016392
Catecholamines	0.47089	0.13193	3.5694	0.070313
Hunt & Hess	0.16144	0.50963	0.31677	0.78143
WFNS	0.38709	0.13474	2.8729	0.10281
VLFO.Ratio	5.525	0.77724	7.1084	0.019221

Table S5 linear regression model evaluating the relationship between Age, Sex, Deep Sedation, Catecholamine, Hunt&Hess, WFNS and VLFO.Ratio with the Glasgow Outcome Scale (GOS) after 12 months. VLFO.ratio appears to be an independent predictor of GOS after 12 months ($p = 0.0050$), even when controlling for possible confounders included

in the model. The model shows a high fit ($R^2 = 0.999$, Adjusted $R^2 = 0.996$), with a highly significant overall effect (F-statistic = 292, $p = 0.003$).

**Variable**	**Estimate**	**SE**	**tStat**	**pValue**
(Intercept)	5.0038	0.64754	7.7274	0.016338
Age	0.0060	0.01407	0.4267	0.71113
Sex	-1.6639	0.21096	-7.8872	0.015698
Deep Sedation	2.2730	0.11112	20.455	0.002382
Catecholamines	-0.0046	0.08198	-0.0556	0.96074
Hunt & Hess	1.7543	0.45645	3.8435	0.061514
WFNS	-0.5018	0.18836	-2.6642	0.11673
VLFO.Ratio	6.7224	0.47995	14.006	0.005059
